# Glaucoma Patients' Trust in the Physician

**DOI:** 10.1155/2009/476726

**Published:** 2009-10-07

**Authors:** Kelly W. Muir, Cecilia Santiago-Turla, Sandra S. Stinnett, Leon W. Herndon, R. Rand Allingham, Pratap Challa, Paul P. Lee

**Affiliations:** Department of Ophthalmology, Duke University Eye Center, Durham, NC 27710, USA

## Abstract

*Objectives*. To describe glaucoma patients' trust in the physician and to test the hypothesis that increased interpersonal trust is associated with increased medication adherence. *Methods*. One hundred ninety-five subjects with open-angle glaucoma seen by multiple glaucoma subspecialists participated in a cross-sectional patient survey and concomitant chart review which included a test of health literacy and the Trust in Physician Scale (TPS), a scale from 1–100, with 100 indicating greatest trust. Charts were reviewed for visual acuity and visual field results. Subjects' pharmacies were contacted to ascertain medication refill rates over the preceding six months. *Results*. TPS scores ranged from 57.5 to 100, 78.7 ± 8.4 (mean ± SD,) median 75.0. When age, race, gender, baseline visual acuity and visual field status, education level, and literacy status were considered, only race was associated with TPS. Caucasians expressed slightly higher levels of trust (*n* = 108; TPS 80.1 ± 8.2) than non-Caucasians, (*n* = 87 (82 Africans Americans); TPS 77.1 ± 8.4; *P* = .012). TPS score was not associated with refill rates (*P* = .190). *Conclusions*. Trust in physician is generally high in this group of glaucoma patients but varies slightly by race. Trust in physician was not associated with glaucoma medication adherence in this tertiary care population.

## 1. Introduction

Greater patient trust in his or her physician is associated with greater patient satisfaction [1] and decreased likelihood of changing physicians [2]. Patients who express greater trust in their primary care physician report increased adherence with their medical regimen [3]. 

 The physician-patient relationship is complex and, most likely, many factors contribute to the level of trust. In the primary care setting, greater length of time in the physician-patient relationship and Caucasian race are associated with greater trust [4, 5]. In addition to race, better baseline health status is associated with increased trust in patients seen by medical subspecialists [6]. 

 As in the primary care setting, patients with glaucoma often see their eye care provider frequently over many years. Similar to the primary care management of chronic diseases such as diabetes and hypertension, the management of glaucoma depends largely on patient self-medication. Whether measured by pharmacy claims data [7, 8], self-report [9, 10], or medication monitor [11], adherence to glaucoma medication is poor. Factors found to contribute to nonadherence include more frequent [12, 13] and complex [14] dosing, situational factors such as competing activities [14], forgetfulness [9], poor disease knowledge [15, 16], and poor health literacy [17]. It has been suggested that elements of the patient-physician relationship may also play a role in patients' adherence to glaucoma treatment [18], and, indeed, Friedman and colleagues found that patients who exhibited a more passive learning style were less likely to adhere to their medications [15]. With these thoughts in mind, we sought to characterize the level of trust in the glaucoma patient-physician relationship, assess the contribution of potential explanatory variables such age, race, gender, and health literacy, and test the hypothesis that greater trust in physician is associated with greater medication adherence. 

## 2. Methods

The study, approved by the Duke University Institutional Review Board, was designed as a cross-sectional patient survey and concomitant chart review. Potential subjects were recruited from the Glaucoma Service of Duke University Eye Center. Each subject was cared for by one of four glaucoma subspecialists. Subjects were included if they had a diagnosis of open-angle glaucoma, and visual field tests were present in the medical record. Subjects who refused to participate or scored less than 18 on the Minimental State Exam (MMSE), a measurement of cognitive status [19], were excluded. From July 2000 through June 2001, 209 potential subjects were approached in the Duke University Eye Center while waiting to see a glaucoma specialist and asked to participate in the survey. Informed consent was obtained for survey participation as well as for review of the medical record. All subjects were approached and all surveys were conducted by the same investigator (CST). The survey included questions relating to demographic data (self-reported race and level of education completed), the MMSE, the Rapid Assessment of Adult Literacy in Medicine (REALM; a word recognition test of functional health literacy) [20], and the Trust in Physician Scale (TPS). The TPS is an 11-item, self-administered questionnaire scored 1–100, with 100 indicating greatest trust. Items included in the TPS were derived from patient interviews and similar testing instruments. Items are answered in a 5-point Likert format. Internal reliability is excellent (Cronbach alpha = 0.90) [21] and test-retest reliability has been validated [3]. The specific items included in the TPS are included in Table 1.

The medical record was reviewed for visual field results and prescribed medication. With informed consent, each subject's pharmacy was contacted to obtain refill data from the previous six months. Each request bottle of medication for each prescribed eye drop was considered one refill. Refill rates have been shown to be a reasonable measure of medication adherence [22, 23] and have been utilized in the ophthalmic literature [13, 17]. 

## 3. Statistical Methods

Initially, descriptive statistics were obtained, (means, standard deviations, medians for continuous data, and frequencies and percentages for categorical data). The relationship between TPS score and demographic (age, sex, race, education, and REALM) and vision variables (visual acuity and mean deviation of visual field) was assessed in a univariable fashion using either a analysis of variance (for categorical predictors) or linear regression (for continuous predictors). All statistical analyses were performed using SAS E-Guide Version 4 for Windows (SAS Institute, Cary, NC). Two-sided *P*-values at the standard .05 level were used to determine statistical significance. 

## 4. Results

Of the 209 subjects approached, nine declined to participate in the complete survey, including seven women and two men, six blacks, two whites, and one subject of unknown race. Five subjects did not meet the MMSE criteria with scores of three, five and 10, 13, and 15. These subjects were all men ranging in age from 71 to 90 years and included two whites, two blacks, and one subject of unknown ethnicity. Characteristics of the 195 subjects who completed the survey are presented in Table 2.

### 4.1. Trust

**Figure 1:**
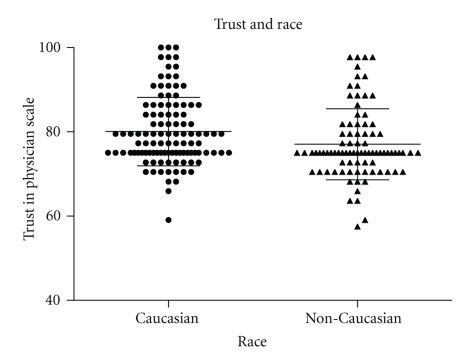
Scores on the Trust in Physician Scale (TPS), a test of interpersonal trust in the physician from the patients' perspective, were plotted for Caucasian and for African American subjects. On the 0 to 100 scale, mean TPS score for the 108 Caucasian subjects was 80.1 ± 8.2 (mean ± SD) which was significantly higher than the mean TPS score for the 87 non-Caucasians, TPS 77.1 ± 8.4; *P* = .012. The dark bars represent mean scores and whiskers indicate SD.

On a scale of 1–100, TPS scores of all subjects ranged from 57.5 to 100, with a mean score of 78.7 ± 8.4 (mean ± SD), and median score of 75.0. When age, race, gender, baseline visual acuity and visual field status, education level, and literacy status (REALM) were considered, only race was associated with TPS. Caucasians expressed higher levels of trust in the physician (*n* = 108; TPS 80.1 ± 8.2) than non-Caucasians (*n* = 87; TPS 77.1 ± 8.4; *P* = .012). Of the 87 non-Caucasians in the study, 82 subjects were African-American (see Figure 1).

The study subjects represent the patients of four physicians. Considering all physicians together, there was a statistically significant difference in the levels of trust reported according to the treating physician (analysis of variance, Kruskal-Wallis *P* = .03). After adjusting for multiple comparisons, however, there was not a significant difference between trust levels for any two physicians, except for between the two physicians with least number of subjects (10 subjects, TPS score 85.9 ± 10.3 and 17 subjects, TPS score 75.8 ± 7.8; Dunn's multiple comparison test, *P* < .05). 

### 4.2. Adherence

**Figure 2:**
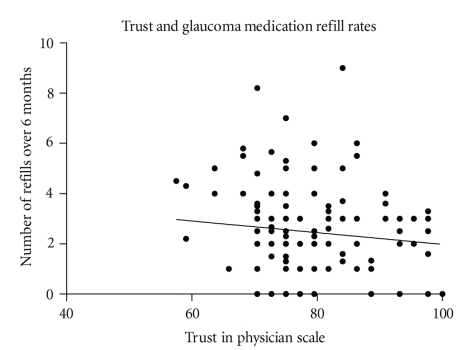
Number of refills for glaucoma medications requested by the 142 subjects for whom such data were available were plotted against scores on the Trust in Physician Scale. Little of the variability in refill rates, a surrogate marker for medication adherence, is explained by trust in this model (*R* ^2^ = 0.01, *P* = .190.)

One hundred and sixty-two of the 195 subjects were prescribed glaucoma medications. Of these, we did not receive a response from the pharmacies for 20 subjects. Thus, adherence was assessed in 142 subjects. The mean number of refills requested over six months for each prescribed medication was 2.5 (standard deviation 1.8; range 0–9). There was no association between TPS score and refill rates (*P* = .190). The correlation of trust score to refill rates was −0.011 (Pearson correlation coefficient). The authors estimated that a sample size of 844 would be needed to detect that this correlation was significantly different from zero (two-sided Fisher's exact test); see Figure 2.

## 5. Discussion

We examined the level of trust in the physician expressed by a group of subjects with glaucoma cared for by several glaucoma subspecialists in an academic practice. We found that trust was generally high with a median score of 75 on the testing instrument scaled 1–100. Of age, race, gender, literacy status, level of spiritual belief and activities, and baseline visual acuity and visual field status, only race was associated with the level of trust. Caucasians expressed slightly greater levels of trust than non-Caucasians, the majority of whom were African American. Similar racial differences in levels of trust in the patient-physician relationship have been described previously [4–6, 24]. We did not examine the relationship between racial concordance (i.e., physician and patient of same race) and trust. Others have found that racial concordance is associated with improved communication in the patient-physician interaction [25, 26] which might lead to improved levels of trust. 

 Other studies have shown an association between increased trust in physician and self-reported adherence to treatment regimen [3]. We did not measure self-reported adherence but rather refill rates as a surrogate marker of medication adherence, and we did not find such an association. One could argue that patients who express greater trust in their physicians are also likely to report better treatment adherence in a, perhaps unconscious, attempt to please the physician, regardless of the actual level of medication adherence. Using refill rates as an adherence marker should avoid this confounder. Additionally, a physician might assume that if he or she has a trusting patient-physician relationship that the patient is more likely to adhere to the medical regimen, but our study does not support this assumption. The fact that we did not find an association between trust and adherence does not mean that a relationship does not exist. Little variation in levels of trust, methodological problems with measuring medication adherence, and a small sample size may have all contributed to the lack of a statistically significant association. Nevertheless, the high levels of trust and relatively poor levels of adherence (both excess and insufficient request for mediation refills) do raise interesting questions. In this same group of subjects, we reported that poor health literacy is associated with decreased medication adherence [17]. Perhaps even if a patient fully trusts his or her physician and the management recommendations, if the patient is not prepared to navigate the healthcare system, treatment adherence suffers. 

 The generalizability of this study is limited by its sample size. Furthermore, all subjects were seen by glaucoma subspecialists in a tertiary care; academic setting and results are thus likely not applicable to all glaucoma patient-physician relationships. In primary care, it has been found that trust increases with the duration of the patient-physician relationship [5, 27, 28]. We did not investigate the duration of the relationship between the patients and physicians in our study, but because of the referral nature of the academic setting, the relationships might be shorter than the average relationships for a general ophthalmologist and his or her patients. We also measured trust among a group of patients who agreed to participate in a research study, which may have excluded distrustful patients from the sample (although less than three percent of potential subjects declined to participate). The academic setting may have affected the measured racial differences in trust. The history of medical research contains many episodes of abuse against African Americans [29]; this history might influence measures of trust more in the research environment than in the general practice. 

 The measurement of medication adherence is problematic. Although using refill rates rather than self-reported medication adherence provides more objectivity, limitations persist. The medical record did not reflect sample medications provided in clinic. If a subject received samples, his or her calculated refill rate would underestimate medication adherence. Conversely, a subject may have requested more bottles than he or she actually used due to travel plans, physical difficulties administering drops, or poor understanding of the prescribed dosing regimen. Unfortunately, all measures of medication adherence, with the possible exception of directly observed therapy, are flawed. Neither refill rates nor electronic medication monitors capture the true percentages of prescribed doses that are successfully instilled into a patient's eye. Nonetheless, refill rates are associated with other measures of medication adherence [23] and offer one perspective, however limited, on how patient's adhere to the prescribed treatment regimen. 

 Even if trust is not a major factor in adherence, evidence from other studies (and not assessed in this study) suggests that improved trust is associated with improved patient satisfaction [1]. In this study, the only characteristic associated with trust was race. Racial disparities continue to exist in eye care. Bilateral blindness is twice as common in African Americans versus Caucasians [30]. Hopefully, a better understanding of the patient-physician relationship will help explain why such inequities persist. Longitudinal studies of the clinical outcomes associated with various parameters of the patient-physician relationship, such as trust, are needed. If we accept that trust is indeed important, literature provides suggestions for how trust can be improved. Keating and colleagues described elements of the clinical encounter associated with more trust in medical subspecialists. The elements included the feeling that the patient spent as much time as he or she wanted with the specialist and that the specialist listened to the patient's concerns [6]. Although time is at a premium for all providers, time, or its attendant expression of concern, may be the crucial element in fostering the trusting relationship to which we aspire.

## Figures and Tables

**Table 1:** Trust in physician scale.

(1)	I doubt that my doctor really cares about me as a person.
(2)	My doctor is usually considerate of my needs and puts them first.
(3)	I trust my doctor so much I always try to follow his/her advice.
(4)	If my doctor tells me something is so, then it must be true.
(5)	I sometimes distrust my doctor's opinions and would like a second one.
(6)	I trust my doctor's judgments about my medical care.
(7)	I feel my doctor does not do everything he/she should about my medical care.
(8)	I trust my doctor to put my medical needs above all other considerations when treating my medical problems.
(9)	My doctor is well qualified to manage (diagnose and treat or make an appropriate referral) medical problems like mine.
(10)	I trust my doctor to tell me if a mistake was made about my treatment.
(11)	I sometimes worry that my doctor may not keep the information we discuss totally private.

**Table 2:** Characteristics of 195 subjects with open angle glaucoma and factors associated with trust in physician.

Variable	*N* (%)	Trust in Physician*	**P-value
		Mean (SD); median (1–100, least to most)	
Race			

White	108 (55)	80.1 (8.2); 78.4	**.012 **
Black	82 (42)	77.1 (8.4); 75.0	
Asian/Pacific Islander	2 (1)		
Latino	2 (1)		
Unknown	1 (.5)		

Gender			

Male	79 (41)	77.9 (7.5); 75.0	.241
Female	116 (59)	79.3 (8.9); 77.2	

Age (years)			

≤65	56 (28)	77.7 (7.5); 75	.750
66–73	43 (22)	80.3 (8.7); 77.3	
74–80	51 (26)	77.7 (8.6); 75	
>80	45 (23)	79.8 (8.7); 77.3	

Visual Field^*⋀*^ in Worse Eye		−0.05 (0.08)	.567
slope (standard error) (*N* = 110)			
Visual Field^*⋀*^ in Better Eye		−0.07(0.09)	.454
slope (standard error) (*N* = 110)			
Visual Acuity in Worse Eye		0.46 (0.76)	.543
slope (standard error)			
Visual Acuity in Better Eye		−3.43 (4.61)	.458
slope (standard error)			

Education			

Did not complete high school	49 (25)	76.7 (6.1); 75.0	.054
High school graduate	145 (75)	79.4 (9.0 : 75.0)	

Health literacy (REALM^*⋀⋀*^ score)			

≤8th grade level	100 (52)	77.7 (7.4); 75.0	.086
≥9th grade level	94 (48)	79.8 (9.2); 77.3	

*Based on Trust in Physician 11-item single-score scale with 1 indicating least and 100 indicating most trust.

**P-values based on Wilcoxon rank sum test for comparison among categories of variables or *t*-tests for slopes).

^*⋀*^Mean deviation in the visual field.

^*⋀⋀*^REALM: Rapid Assessment of Adult Literacy in Medicine.
